# Transcriptome Analysis and Characterization of Two Detoxification Genes Responding to Imidacloprid in *Aphis spiraecola*

**DOI:** 10.3390/insects17090973

**Published:** 2026-09-21

**Authors:** Meng Zhang, Chunping Si, Hanjie Liu, Liting Chai

**Affiliations:** School of Food and Strategic Reserves, Henan University of Technology, Zhengzhou 450001, China

**Keywords:** *Aphis spiraecola*, imidacloprid, response to insecticide, transcriptome, cytochrome P450, CarE

## Abstract

*Aphis spiraecola* is a major pest of fruit trees worldwide, causing significant yield reductions and economic losses in apple production. Chemical control remains the primary and most effective method for managing *A. spiraecola* in the field. Imidacloprid, the first neonicotinoid insecticide, effectively targets piercing–sucking pests. However, the time-dependent response of *A. spiraecola* to imidacloprid exposure has not been well characterized. In this study, we assessed the susceptibility of an *A. spiraecola* population from Zhengzhou to imidacloprid. Enzymatic assays revealed that the activities of cytochrome P450s, glutathione S-transferases (GSTs), and carboxylesterases (CarEs) were significantly increased following imidacloprid treatment. A synergism assay further demonstrated that the P450 inhibitor PBO and the CarE inhibitor TPP significantly enhanced the imidacloprid toxicity against *A. spiraecola*. Transcriptome analysis revealed that LC_50_ imidacloprid treatment induced time-dependent changes in gene expression at 12, 24, and 36 h. A total of 46 P450 genes, 42 esterase genes, and six GST genes were identified in the transcriptome of *A. spiraecola*. Five detoxification enzyme genes, *CYP380C6*, *CYP6CY19*, *GSTS3*, *GSTL*, and *CarE6*, were significantly overexpressed, as confirmed by both transcriptomic and qPCR analysis. Moreover, RNAi-mediated knockdown of *CYP6CY19* and *CarE6* substantially increased aphid sensitivity to imidacloprid compared with the ds*GFP* control group. These results demonstrate that *CYP6CY19* and *CarE6* play an important roles in determining *A. spiraecola* susceptibility to imidacloprid. Our findings provide a theoretical basis for the rational and scientific use of imidacloprid in aphid management.

## Simple Summary

*Aphis spiraecola* is an important pest of fruit trees, and imidacloprid is widely used for its control. However, how this aphid responds to imidacloprid over time and which detoxification genes contribute to its susceptibility remain unclear. In this study, we investigated the time-dependent physiological and molecular responses of an *A. spiraecola* population from Zhengzhou to imidacloprid. Imidacloprid exposure significantly increased the activities of cytochrome P450 monooxygenases, glutathione S-transferases, and carboxylesterases, while inhibitors of P450s and carboxylesterases enhanced imidacloprid toxicity. Transcriptome and qPCR analyses further identified several detoxification genes that were strongly induced after treatment. Functional analysis using RNA interference showed that silencing of *CYP6CY19* and *CarE6* significantly increased aphid susceptibility to imidacloprid. These findings demonstrate that *CYP6CY19* and *CarE6* are important components of the detoxification response to imidacloprid. This study improves our understanding of insecticide responses in *A. spiraecola* and may support more rational insecticide use and sustainable aphid management.

## 1. Introduction

The *Aphis spiraecola* Patch is a polyphagous aphid species belonging to the order Hemiptera and the family Aphididae, found in both temperate and tropical regions [1,2]. This aphid has a wide range of host plants, including apple (*Malus domestica* Borkh.), pear (*Pyrus* spp.), crabapple (*Malus* spp.), hawthorn (*Crataegus pinnatifida* Bunge), apricot (*Prunus armeniaca* L.), plum (*Prunus domestica* L.), *Spiraea* spp., grapefruit (*Citrus paradisi* Macfadyen), *Polyscias crispata* (Bull) Merrill, *P. scutellaria* (Burman) Fosberg, *Vibernum suspensum* Lindley, rough lemon (*C. jambhiri* Lushington), and pineapple orange [*C. sinensis* (L.) Osbeck], making it a significant pest threat to fruit trees [3,4,5]. *A. spiraecola* feeds on the young shoots and underside of leaves, causing the leaves to curl inward or downward. This disrupts photosynthesis and hinders the normal growth of branches [6]. Moreover, the honeydew secreted by *A. spiraecola* promotes the growth of sooty mold on the leaves and fruit, reducing the ability of the plants to intercept radiation during photosynthesis and further decreasing the yield of marketable fruit [7]. In China, *A. spiraecola* begins to damage crops in late April, with peak infestation occurring in May and June, and continuing until the apple harvest in September [8]. Over the past few decades, this aphid has become a widely distributed pest worldwide. Chemical control remains the primary method for managing apple aphid infestations including *A. spiraecola*. However, the prolonged and intensive use of chemical pesticides has led to varying degrees of resistance to commonly used insecticides [9,10,11].

Imidacloprid, the first neonicotinoid insecticide, is widely used to control both sucking and biting insect pests. It is the most popular insecticide globally, owing to its high efficacy, broad-spectrum activity, and low toxicity to mammals. Insects’ nicotinic acetylcholine receptors (nAChRs) are primarily located in the central nervous system, where they function as receptors for acetylcholine, a neurotransmitter essential for synaptic transmission. Imidacloprid targets nAChRs, disrupting nerve signal transmission and blocking the insect’s neural pathways, leading to the accumulation of acetylcholine at the receptors and ultimately causing the insect’s death [12]. Due to its low lipophilicity and excellent plant uptake and translocation properties, imidacloprid remain an effective measure for controlling piercing–sucking pests such as *A. spiraecola*.

Interestingly, during the application of insecticides, in addition to directly killing pests, some insecticidal substances remain in the natural environment. The use of imidacloprid for pest management can result in detectable residues of the parent compound and its metabolites in apple fruits, raising concerns regarding potential dietary exposure and food safety [13]. Due to factors such as plant coverage, natural degradation, and volatilization, these residual pesticides gradually break down to sublethal concentrations [14,15]. Numerous studies have shown that exposing insects to sublethal concentration of insecticides leads to changes in the physiological metabolism. These changes include the induction of detoxification enzymes such as glutathione S-transferase (GST), carboxylesterase (CarE), and cytochrome P450 monooxygenase (P450), along with alterations in the expression of related genes. Two carboxylesterase genes (*CL5401-1* and *CL5480-4*) in *Sogatella furcifera* [16], *CYP6FV12* in *Bradysia odoriphaga* [17], *CYP303A1*, *CYP4C62*, *CYP6BD5*, *GSTS1* and *EST-6* in *Diaphorina citri* [18], 11 P450, two CarE, four GST, and two esterase genes in *Sitobion avenae* [19], *CYP4g15*, *CYP18A1*, *CYP6CY21*, *CYP6DA1*, *CYP6DA2*, *CYP4CJ1*, *CYP4CJ2* and *CYP380C6* in *Aphis craccivora* [20], *CYP6QE1* and *CYP6FV21* in *Bradysia odoriphaga* [21], *NlGSTs1*, *NlGSTs2* and *NlGSTe1* in *Nilaparvata lugens* [22], *CYP6CY7* in *Aphis glycines* [23], *CYP303A1*, *CYP4DE1*, *CYP3184-fragment1*, *CYP3939A2*, *CE-1*, *CE-2*, *CE-3* and ABC genes in *Amrasca biguttula* [24], were up-regulated under imidacloprid treatment with different concentrations. Interestingly, the genes that respond to imidacloprid differ across various insect species. However, there have been no reports yet on the effects of median lethal concentration of imidacloprid on the apple aphid (*A. spiraecola*).

In this study, bioassay was conducted to evaluate the susceptibility of *A. spiraecola* to imidacloprid. Based on the transcriptome database, we investigated the response of *A. spiraecola* after exposure to the median lethal concentration (LC_50_) of imidacloprid at different time points. Furthermore, the enzyme activities of GST, CarE and P450 were analyzed among control and imidacloprid treatment groups. Then, the expression patterns of GST, CarE and P450 genes were performed in *A. spiraecola* after exposure to imidacloprid. RNA interference (RNAi) was applied to investigate the role of these significantly up-regulated detoxification genes in the susceptibility of *A. spiraecola* to imidacloprid. Our study combines insecticide use with RNAi technology to offer mechanistic insights that can contribute to the development of innovative pest management strategies.

## 2. Materials and Methods

### 2.1. Aphids

The *A. spiraecola* population was obtained from apple orchard in Zhengzhou, Henan Province, China and subsequently maintained on apple seedlings under standard conditions of at 25 ± 1 °C, 70 ± 5% relative humidity, and a photoperiod of 16:8 h (light:dark). Apple seedlings and aphids were enclosed in cages measuring 100 × 30 × 30 cm, covered with mesh gauze (100 mesh), to prevent aphid escape.

### 2.2. Bioassays

The insecticide (imidacloprid, CAS: 138261-41-3) tested in this study were purchased from Shanghai Ampu Experiment Co., Ltd. (Shanghai, China) with a purity of ≥95%. The toxicity of imidacloprid to *A. spiraecola* population was assessed using a leaf-dip method, as modified from Wang et al. [11]. Briefly, imidacloprid was prepared at a concentration of 10 g/L in acetone and diluted in water containing 0.1% Triton X-100 to form a series of solutions (1.88, 3.75, 7.50, 15.00, and 30.00 mg/L). The control group was provided with water that contained 0.1% Triton X-100 and 0.01% acetone. Apple leaves with apterous adult aphids were treated in the solutions for 10 s, with residual droplets absorbed by sterile filter paper. The treated leaves were then placed in a cylindrical plastic box with a diameter of 10 cm and maintained under the previously described conditions. The mortality was recorded 24 h post-treatment for approximately 30 aphids at five different concentrations, tested in three replicates.

### 2.3. Determination of the Enzyme Activities of GST, CarE and P450

The activity of the three detoxifying enzymes (P450, GST, and CarE) was measured following the method of Su et al. [25] and Tang et al. [1] using assay kits purchased from Suzhou Grace Biotechnology Co., Ltd (Suzhou, China). Thirty apterous adult aphids were individually homogenized in 1 mL of pre-chilled sodium phosphate buffer on ice to prepare the enzyme source. The homogenate was then centrifuged at 12,000× *g* for 30 min at 4 °C. The supernatant was carefully collected and subjected to a second centrifugation at 12,000× *g* for an additional 10 min at 4 °C. The total protein concentration in the enzyme extract was measured using the Bradford method [26], with BSA as the standard, and the optical density (OD) was recorded at 562 nm. The resulting clear supernatant was utilized for enzyme activity.

After mixing the enzyme solution with p-nitroanisole and NADPH, the initial OD (A0) was measured at 405 nm using an iMark Microplate Reader (Bio-Rad, Hercules, CA, USA). Following this, the mixture was incubated at 37 °C for 30 min, after which the OD (A1) was measured again. The formula for calculating enzyme P450 activity presented by absorbance is P450 activity = ((A1 − A0 + 0.0058)/2.4575 × 1000 × V)/(V × Cp)/T, where T is the absorbance period (30 min), and Cp is the concentration of sample protein, and V is the sample volume. To assess GST activity, the reaction involved the substrate 1-chloro-2,4-dinitrobenzene (CDNB) and reduced glutathione (GSH) in combination with the enzyme solution. The change in absorbance was recorded at 340 nm over a period of 5 min at 37 °C. The equation used to determine GST activity based on absorbance is as follows: GST activity = (ΔOD × V)/(ε × T × L × E). In this formula, ΔOD represents the change in absorbance at 340 nm over a 5 min interval, V is the total reaction volume of 210 µL, ε is the extinction coefficient of the product at 9.6 mL/(μmol·cm), T is the duration of the measurement (5 min), L is the optical path length of the solution at 1 cm, and E denotes the protein concentration in the reaction system. To assess CarE activity, the reaction involved the chromogenic agents and α-naphthyl acetate in combination with the enzyme solution. The change in absorbance was recorded at 450 nm over a period of 3 min at 37 °C. The equation used to determine CarE activity based on absorbance is as follows: CarE activity = ΔOD ÷ (V × Cpr) ÷ T × D. In this formula, ΔOD represents the change in absorbance at 450 nm over a 3 min interval, V is the total reaction volume of 30 µL, Cpr is the concentration of sample protein, T is the duration of the measurement (3 min), and D represents the dilution factor. The enzyme activity assays were conducted with three independent biological replicates.

### 2.4. Synergism Bioassay

For the enzyme inhibitor synergism assay, piperonyl butoxide (PBO), triphenyl phosphate (TPP), and diethyl maleate (DEM) were dissolved in acetone. Based on previously described methods with minor modifications [27], aphids were individually pretreated with 100 mg/L TPP, PBO, or DEM 2 h before insecticide exposure. Subsequent insecticide treatments were performed using the leaf-dipping bioassay protocol. The treated aphids were maintained on young leafy apple shoots under controlled conditions of 25 ± 1 °C, 70–80% relative humidity, and a 16 h light:8 h dark photoperiod. Each treatment included four replicates, with 30 adult aphids per replicate. Mortality was recorded after 24 h.

### 2.5. Transcriptome Data Analysis

To investigate the temporal response of *A. spiraecola* to imidacloprid exposure at the LC_50_ concentration, mortality was recorded at different time points (12, 24, 36, 48, 60 and 72 h) following treatment. Based on the observed mortality dynamics, three representative post-treatment time points (12, 24, and 36 h) were selected for transcriptomic analysis to characterize temporal gene expression patterns in response to imidacloprid. *A. spiraecola* treated with water containing 0.1% Triton X-100 and 0.01% acetone was used as the control group. Each treatment consisted of three biological replicates. Total RNA was extracted using TRIzol reagent (Vazyme Biotech Co., Ltd., Nanjing, China). The quality and concentration of RNA were evaluated using a Qubit 2.0 Fluorometer (Thermo Fisher Scientific, Shanghai, China) and agarose gel electrophoresis. For cDNA library construction, approximately 1 μg of high-quality RNA from each sample was employed. Poly(A) + mRNA was enriched using oligo(dT) magnetic beads (Invitrogen, Carlsbad, CA, USA), followed by fragmentation and reverse transcription into cDNA. The library underwent end repair, A-tailing, and adapter ligation, with quality assessment performed using an Agilent 2100 Bioanalyzer (Agilent Technologies, Santa Clara, CA, USA). Libraries were sequenced on the Illumina NovaSeq platform (Biomarker Technologies, Beijing, China), generating 150 bp paired-end reads. The raw data were processed with Trimmomatic v0.39 to remove adapter sequences, low-quality reads, and reads with ambiguous bases, resulting in Clean Data. De novo assembly was performed using Trinity v2.15.1. Clean reads were assembled into contigs and unigenes for subsequent annotation and analysis. Gene expression levels were represented as fragments per kilobase of transcript per million mapped reads (FPKM) for visualization purposes. Differentially expressed genes (DEGs) between imidacloprid treatments and control were identified using DESeq2 v1.34.0 based on gene-level raw counts (|log_2_FC| > 1; Benjamini–Hochberg adjusted *p*-value (padj) < 0.05). Functional enrichment analyses of DEGs were performed with the clusterProfiler v4.3.2 R package.

### 2.6. The Expression Patterns of Up-Regulated GST, CarE and P450 Genes

To verify the differential expression of target genes based on transcriptome analysis, *A. spiraecola* adults were exposed to imidacloprid at concentrations of LC_50_. Surviving aphids were sampled 12, 24, and 36 h after treatment for RNA extraction, as well as a control treatment with 0.01% Triton X-100 and 0.01% acetone. Total RNA was isolated using TRIzol reagent (Vazyme Biotech Co., Ltd., Nanjing, China) and reverse-transcribed into first-strand complementary DNA (cDNA) using the HiScript II Q RT SuperMix Kit (Vazyme Biotech Co., Ltd., Nanjing, China) according to the manufacturer’s protocols.

Quantitative real-time PCR (qRT-PCR) was performed with ChamQ Universal SYBR qPCR Master Mix (Vazyme Biotech Co., Ltd., Nanjing, China) on a LightCycler 480 Real-Time PCR System (Roche, Shanghai, China). The primer sequences are listed in Table A1 in Appendix A. Each qRT-PCR reaction, with a total volume of 20 μL, consisted of 10 μL of SYBR mix, 6.4 μL of double-distilled water, 0.8 μL of each forward and reverse primer, and 2 μL of diluted cDNA template. The thermal cycling conditions were as follows: an initial denaturation at 95 °C for 30 s; followed by 40 cycles of 95 °C for 10 s, 58 °C for 30 s, and 72 °C for 30 s; and concluding with a final extension at 72 °C for 10 min. A melting curve analysis was conducted to ensure the specificity of the amplification.

The relative expression levels of the target genes were normalized against the reference genes *β-actin* and elongation factor 1α (*EF1α*) using the 2^−ΔΔCt^ method [28]. Each treatment comprised three independent biological replicates, and all biological replicates were analyzed in technical triplicate.

### 2.7. RNA Interference

Because cytochrome P450s, glutathione S-transferases (GSTs), and carboxylesterases belong to large multigene families, the sequence specificity of the dsRNA fragments was evaluated before RNAi experiments. The dsRNA target sequences were compared with homologous genes to identify closely related family members with high sequence similarity. To further assess potential off-target effects, the expression levels of the most closely related homologous genes were examined by RT-qPCR after dsRNA-mediated knockdown of each target gene. *A. spiraecola* treated with ds*GFP* was used as a control.

The double-stranded RNA (dsRNA) and quantitative primers were designed using Primer Premier 6.0 (Table A1 in Appendix A), and the dsRNA was synthesized using the T7 RiboMAX™ Express RNAi System (Promega, Madison, WI, USA) according to the manufacturer’s protocols. The concentration and quality of dsRNA were evaluated using an Eppendorf BioPhotometer Plus (Eppendorf, Hamburg, Germany) and analyzed via 1.0% agarose gel electrophoresis. The injection methods for dsRNA were adapted from those outlined by Peng et al. [29]. In summary, 50 nL of dsRNA (10,000 ng/μL) was injected into the thorax segments of adult aphids using a micro-injector (Märzhäuser, Wetzlar, Germany) equipped with a microglass needle created with a P-97 Micropipette Puller (Sutter Instrument Co., Novato, CA, USA). In general, the mortality of *R. padi* following dsRNA injection did not exceed 20%. At 24, 48, 72, and 96 h post-treatment, ten surviving adult aphids from each treatment were randomly collected to evaluate RNAi efficiency. The control group received injections of double-stranded green fluorescent protein (ds*GFP*). To ensure result reliability, three biological replicates were performed for each sample.

### 2.8. Bioassays After RNAi

To further investigate the role of differentially expressed genes in the response of *A. spiraecola* to imidacloprid, the susceptibility of *A. spiraecola* to the insecticide was assessed using the leaf-dipping bioassay described previously, following RNAi treatment. Based on the RNAi efficiency determined previously, imidacloprid bioassays were conducted at the time point when silencing of the target gene reached its maximum after dsRNA injection. Approximately 30 apterous adult aphids from each treatment group were exposed to two different concentrations (LC_30_ and LC_50_) of imidacloprid in *A. spiraecola*. Mortality was assessed 24 h after treatment. Control groups comprised *A. spiraecola* injected with ddH_2_O or ds*GFP*. To ensure result reliability, four biological replicates were performed for each sample.

### 2.9. Statistical Analysis

Bioassay data were processed using DPS software v7.05 (Zhejiang University, Hangzhou, China) to calculate LC_20_, LC_30_ and LC_50_ values with 95% confidence limits (CL). Statistical analysis of all experimental data was conducted using SPSS v.20 (IBM-SPSS, NY, USA). Mortality percentages were log-transformed to satisfy the normality and homoscedasticity assumptions necessary for analysis. The significance of differences between two samples was assessed using a 2-tailed Student’s *t*-test, while comparisons among more than two groups were made using one-way analysis of variance (ANOVA), followed by Tukey’s HSD test for pairwise comparisons.

## 3. Results

### 3.1. Susceptibility of A. spiraecola to Imidacloprid

The probit analysis of imidacloprid toxicity in *A. spiraecola* is summarized in Table 1. The LC_20_, LC_30_ and LC_50_ values for imidacloprid in the *A. spiraecola* population were 7.45 mg/L (95% Confidence limit, 6.37–8.70), 9.35 mg/L (95% Confidence limit, 8.03–12.21), and 13.61 mg/L (95% Confidence limit, 11.33–17.74), respectively. The toxicity regression equations and slope ± SE of imidacloprid on *A. spiraecola* were Y = 1.36 + 3.21*X and 3.21 ± 0.42, respectively.

### 3.2. Effect of Imidacloprid on the Detoxification Enzymes of A. spiraecola

**Figure 1:**
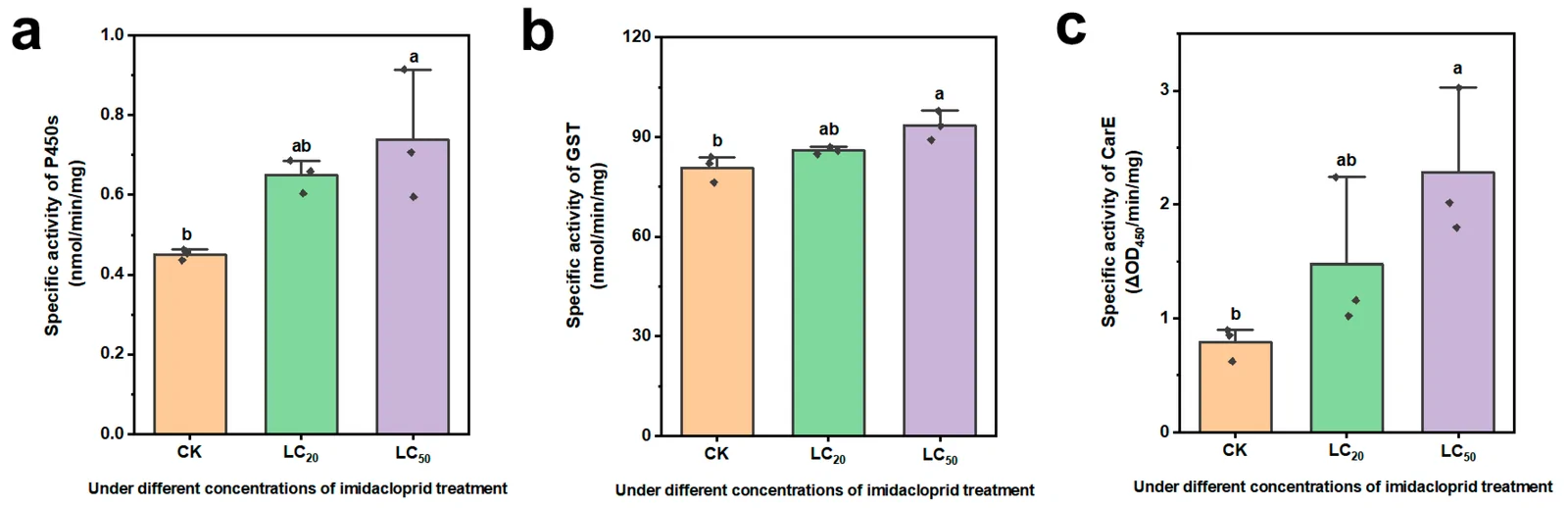
The enzyme activities of P450 (**a**), GST (**b**) and CarE (**c**) in *Aphis spiraecola* after imidacloprid treatment. Data are presented as the mean ± SEM. Bars labeled with different lowercase letters indicate significant differences among treatments, as determined by one-way ANOVA followed by Tukey’s multiple comparison test (*p* < 0.05).

Imidacloprid exposure enhanced the activities of three detoxification enzymes (P450, GST and CarE) in *A. spiraecola*. P450 activity increased to 0.65 ± 0.024 and 0.74 ± 0.094 nmol/min/mg under imidacloprid treatment with concentrations of LC_20_ and LC_50_, respectively, representing 1.44- and 1.64-fold increases compared with the control (F = 6.87; *df* = 2, 6; *p* = 0.028; Figure 1a). GST activity was significantly elevated under LC_50_ imidacloprid treatment, reaching 93.44 ± 2.53 nmol/min/mg, which was 1.16-fold higher than that in the control, whereas LC_20_ imidacloprid treatment resulted in a 1.06-fold increase (F = 10.19; *df* = 2, 6; *p* = 0.012; Figure 1b). Carboxylesterase activity increased in a concentration-dependent manner, with activities of 0.79 ± 0.086, 1.47 ± 0.39, and 2.28 ± 0.38 ΔOD_450_/min/mg protein in the control, LC_20_, and LC_50_ groups, respectively. These values represented 1.87- and 2.89-fold increases under LC_20_ and LC_50_ exposure, respectively, compared with the control (F = 5.60; *df* = 2, 6; *p* = 0.042; Figure 1c).

### 3.3. Synergistic Effects of PBO, TPP and DEM on the Mortality of A. spiraecola

**Figure 2:**
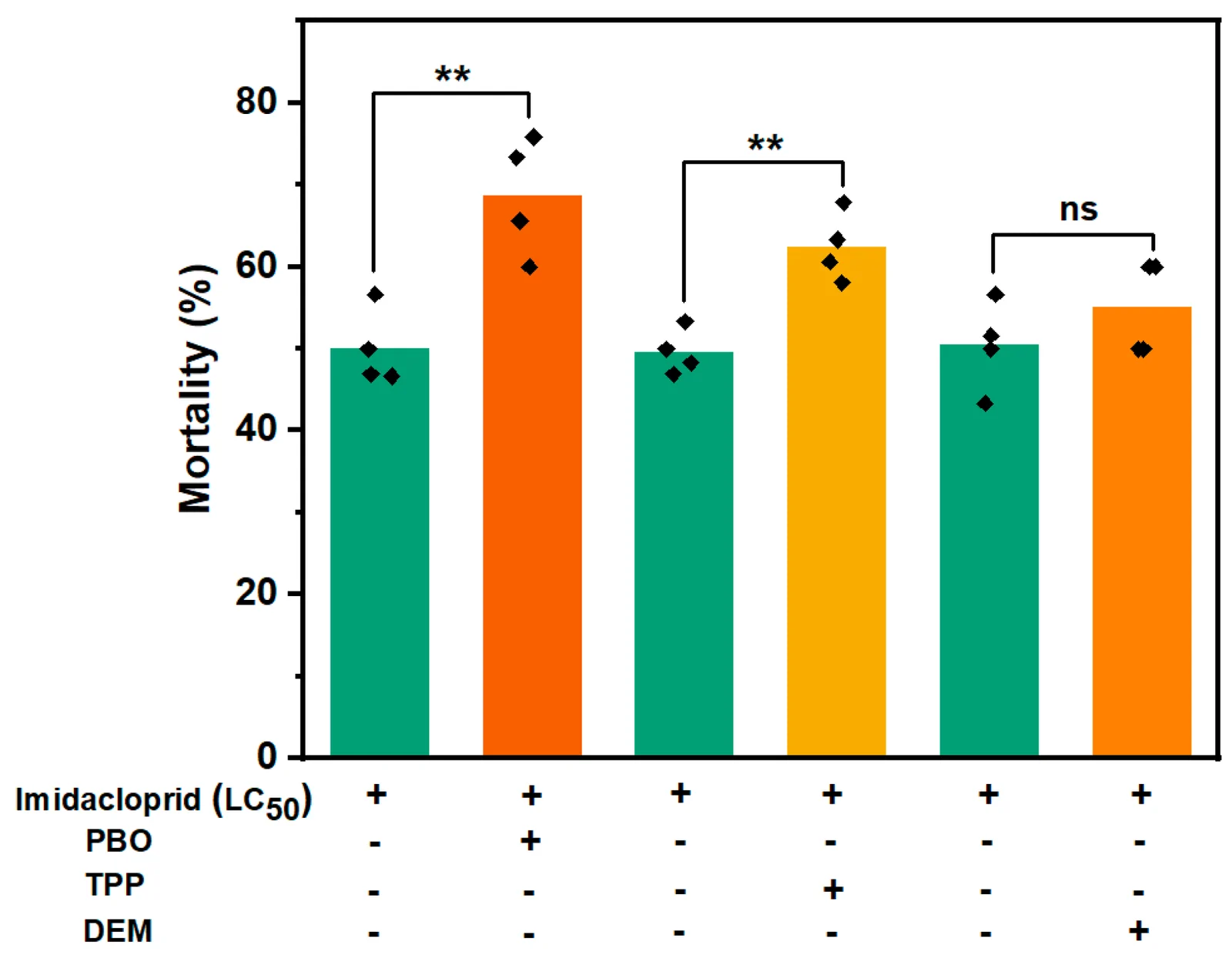
Effects of the synergists PBO, TPP, and DEM on the mortality of *Aphis spiraecola* following median lethal imidacloprid exposure. Double asterisks (**) denote significant differences compared with the control group at *p* < 0.05 and *p* < 0.01, respectively. “ns” indicates no significant difference.

To assess the relative contributions of P450, GST, and CarE to the response of the apple aphid to imidacloprid, the commonly used synergists PBO, DEM, and TPP, which inhibit P450s, GSTs, and esterases, respectively, were individually combined with imidacloprid. Treatment with PBO or TPP significantly enhanced the toxicity of imidacloprid against *A. spiraecola* (Figure 2).

Under the median lethal concentration of imidacloprid (LC_50_) exposure, mortality increased by 1.37-fold compared with the control, reaching 68.71 ± 3.63% (*t*-test, *p* = 0.005). TPP treatment also significantly increased mortality by 1.26-fold under LC_50_ imidacloprid exposure relative to the control, with mortality reaching 62.47 ± 2.09% (*t*-test, *p* = 0.002). In contrast, DEM treatment showed no significant synergistic effect on imidacloprid-induced mortality in *A. spiraecola*, with mortality rates of 50.40 ± 2.75% and 55.00 ± 2.89% in the DEM-treated and control groups, respectively (*t*-test, *p* = 0.29). These results suggest that P450s and CarEs may play important roles in the response of *A. spiraecola* to imidacloprid exposure.

### 3.4. Transcriptome Analysis

**Figure A1:**
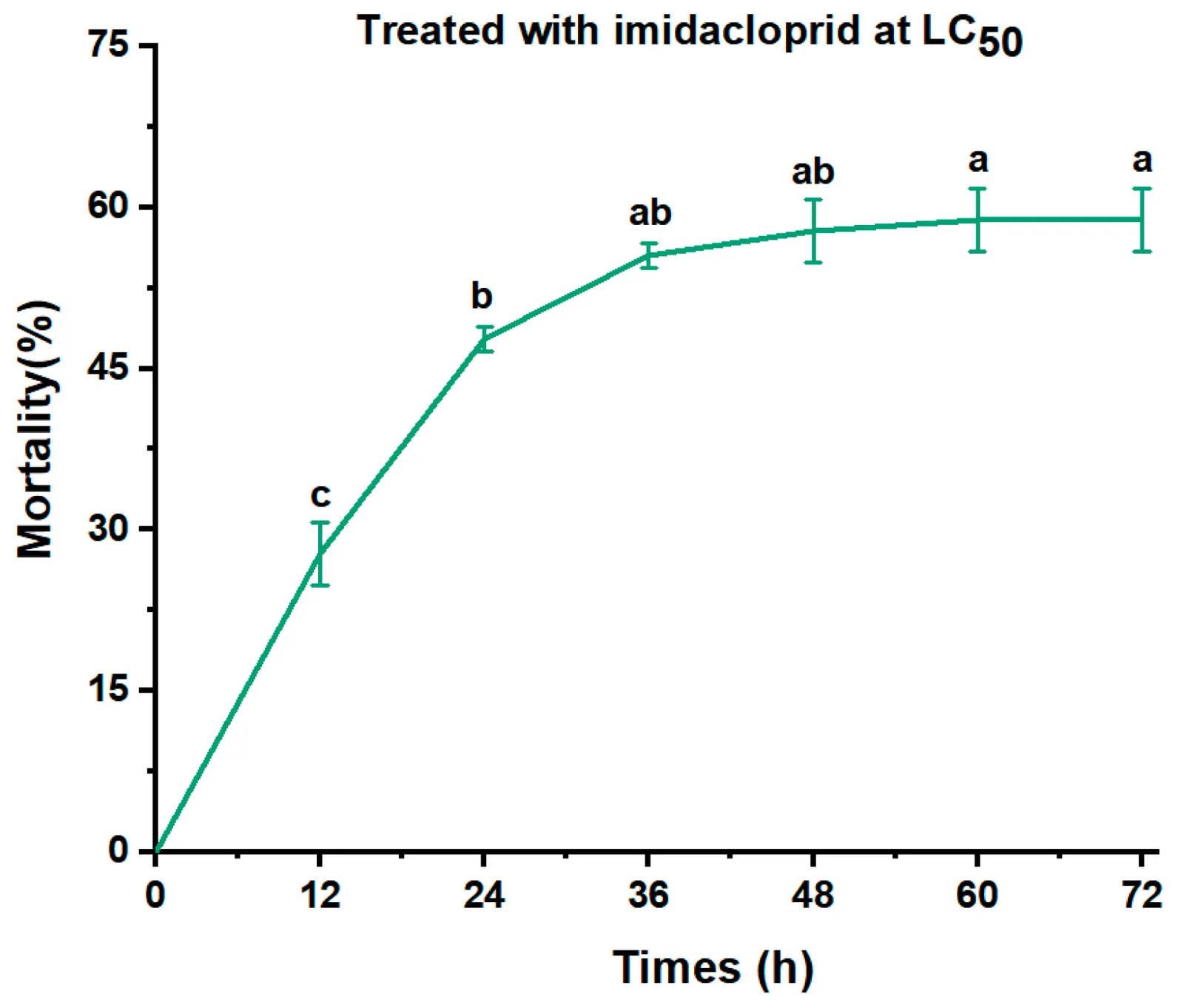
Mortality of *Aphis spiraecola* at different time points following LC_50_ imidacloprid treatment.

Mortality of *A. spiraecola* increased progressively with exposure duration following treatment with imidacloprid at the LC_50_ concentration (Figure A1 in Appendix B). Mortality rose rapidly from 27.78% at 12 h to 47.78% at 24 h and further to 55.56% at 36 h. Thereafter, only a slight increase was observed, with mortality remaining at approximately 58% from 48 to 72 h. These results indicate that imidacloprid-induced mortality increased predominantly during the first 36 h of exposure and subsequently approached a plateau.

Accordingly, 12, 24, and 36 h post-treatment were selected as representative time points to characterize the temporal response of *A. spiraecola* to imidacloprid exposure. To further elucidate the key genes underlying this response, transcriptome analysis was conducted. Twelve independent cDNA libraries (CK_1, CK_2, CK_3, T12_1, T12_2, T12_3, T24_1, T24_2, T24_3, T36_1, T36_2, and T36_3) were constructed from *A. spiraecola* with a mean of 6 Gb of clean data per cDNA library (Table 2). A total of 89.02 Gb of clean reads were obtained, and the percentage of Q30 bases in all samples was not less than 90.07%. The GC contents of the samples ranged from 37.46% to 39.66%. Across the 12 samples, each library generated 21.70–30.34 million clean reads, of which 17.78–25.06 million were successfully mapped to the reference genome. Mapping rates were consistently high (80.92–84.72%), indicating robust sequencing quality and reliable alignment performance (Table 2). The length distribution indicates that most transcripts (35.78%) were longer than 2000 bp, whereas most unigenes (24.72%) were within the 500–1000 bp range. In total, 112,250 transcripts and 59,440 unigenes were obtained, with N_50_ lengths of 3408 bp and 2412 bp and mean lengths of 2020.45 bp and 1345.01 bp, respectively (Table A1).

### 3.5. Differentially Expressed Genes (DEGs)

**Figure 3:**
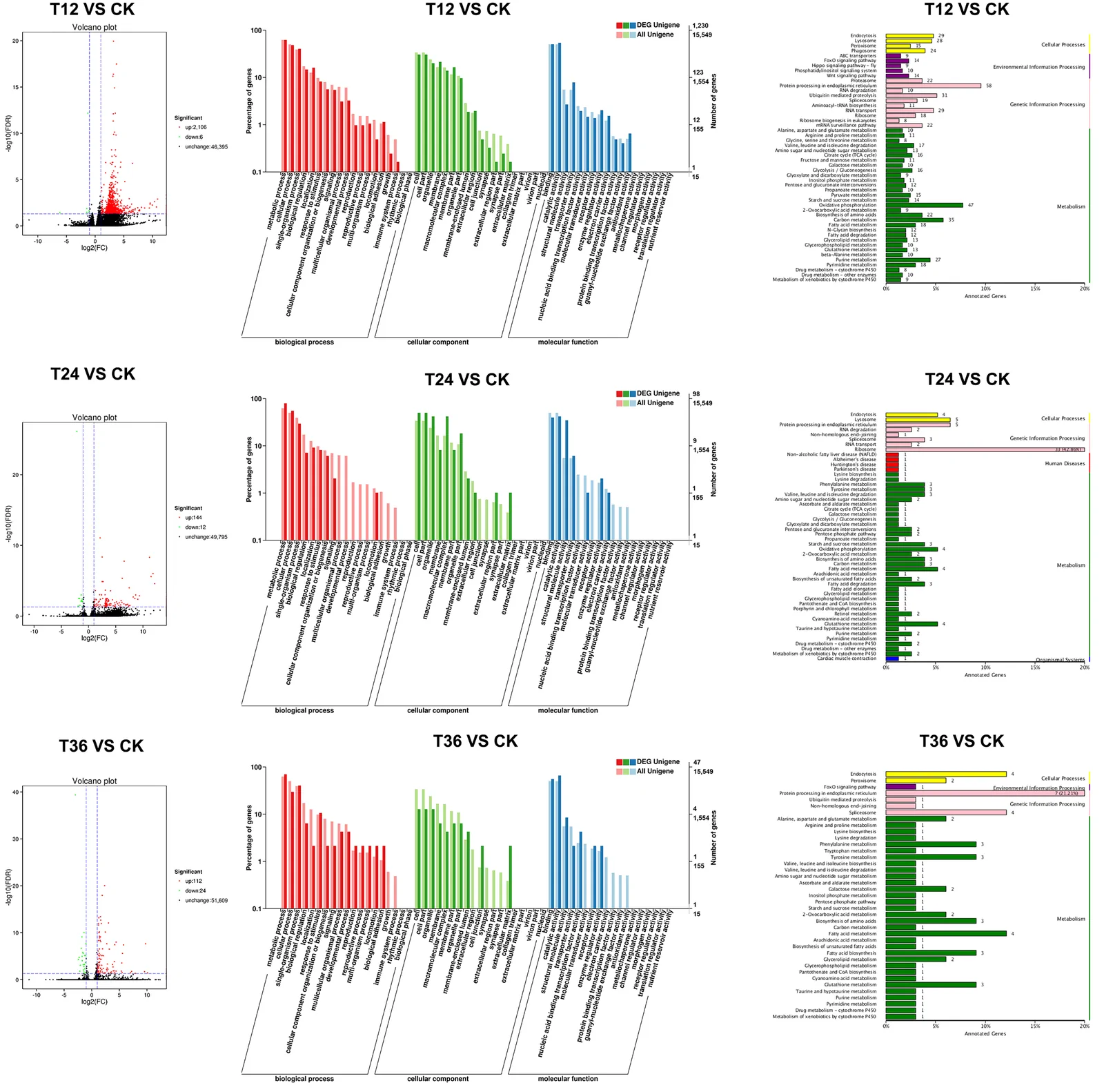
Volcano plots, Gene Ontology (GO) enrichment analysis, and KEGG pathway enrichment analysis of differentially expressed genes (DEGs) in the T12 vs. CK, T24 vs. CK, and T36 vs. CK comparisons.

Based on statistical thresholds (False Discovery Rate (FDR) < 0.05 and |log_2_FoldChange| > 1), differentially expressed genes were identified across various treatment conditions and are summarized in Figure 3. Differential expression analysis identified 2112 DEGs in CK_vs_T12 (2106 up-regulated and 6 down-regulated). In addition, 156 DEGs were detected in CK_vs_T24 (144 up-regulated and 12 down-regulated) and 136 DEGs in CK_vs_T36 (112 up-regulated and 24 down-regulated).

Using NCBI BLAST+ v2.12.0 and HMMER v3.3.2 searches with thresholds of E-value ≤ 1 × 10^−5^ and E-value ≤ 1 × 10^−10^, respectively, 31,314 unigenes were successfully annotated in at least one functional database, accounting for 52.68% of the 59,440 assembled unigenes. Gene Ontology (GO) and KEGG analyses indicated substantial differences in the functional classification of DEGs between the control and imidacloprid-treated groups. Specifically, 15,544 unigenes (26.15%) were assigned to GO terms, whereas 8377 unigenes (14.09%) were mapped to KEGG pathways. Among the GO-annotated unigenes, 10,775 were classified into 4119 functional subclasses spanning the three principal GO categories: biological process (BP), cellular component (CC), and molecular function (MF). In total, 2620 unigenes (16.86%) were assigned to BP terms, 2118 (13.63%) to CC terms, and 10,806 (69.52%) to MF terms (Figure 3).

In the CK_vs_T12 comparison, GO classification showed that the most highly represented BP terms were metabolic process (638 DEGs), cellular process (590 DEGs), and single-organism process (464 DEGs). Within the CC category, the dominant terms were cell (375 DEGs), cell part (375 DEGs), and organelle (247 DEGs). For the MF category, catalytic activity (666 DEGs), binding (622 DEGs), and transporter activity (97 DEGs) were the most abundant terms (Figure 3). KEGG pathway enrichment analysis further revealed that the DEGs were mainly associated with protein processing in the endoplasmic reticulum (58 DEGs), oxidative phosphorylation (47 DEGs), and carbon metabolism (35 DEGs). In addition, several detoxification-related pathways were enriched, including drug metabolism by other enzymes, metabolism of xenobiotics by cytochrome P450, and drug metabolism by cytochrome P450, which contained 9, 9, and 8 DEGs, respectively (Figure 3).

In the CK_vs_T24 comparison, 80, 52, and 91 DEGs were assigned to BP, CC, and MF categories, respectively. KEGG enrichment analysis showed that the most prominently enriched pathway was ribosome (33 DEGs, 4.62%). Detoxification-related pathways were also detected in this comparison, including metabolism of xenobiotics by cytochrome P450, drug metabolism by cytochrome P450, and drug metabolism by other enzymes, which contained 2, 2, and 1 DEGs, respectively. For the CK_vs_T36 comparison, 27, 10, and 42 DEGs were annotated to BP, CC, and MF categories, respectively. KEGG pathway enrichment analysis indicated that these DEGs were mainly enriched in protein processing in the endoplasmic reticulum (7 DEGs) and fatty acid metabolism (4 DEGs). Moreover, detoxification-related pathways, including metabolism of xenobiotics by cytochrome P450 and drug metabolism by cytochrome P450, were also enriched (Figure 3). Overall, the number of DEGs assigned to GO terms was higher in the CK_vs_T12 comparison than in the CK_vs_T24 and CK_vs_T36 comparisons, indicating that T12 induced a broader transcriptional response. The enrichment of pathways related to endoplasmic reticulum protein processing, energy metabolism, carbon metabolism, fatty acid metabolism, and xenobiotic detoxification suggests that the response of *A. spiraecola* to imidacloprid treatment involves coordinated changes in cellular metabolism, protein homeostasis, and detoxification processes.

**Figure A2:**
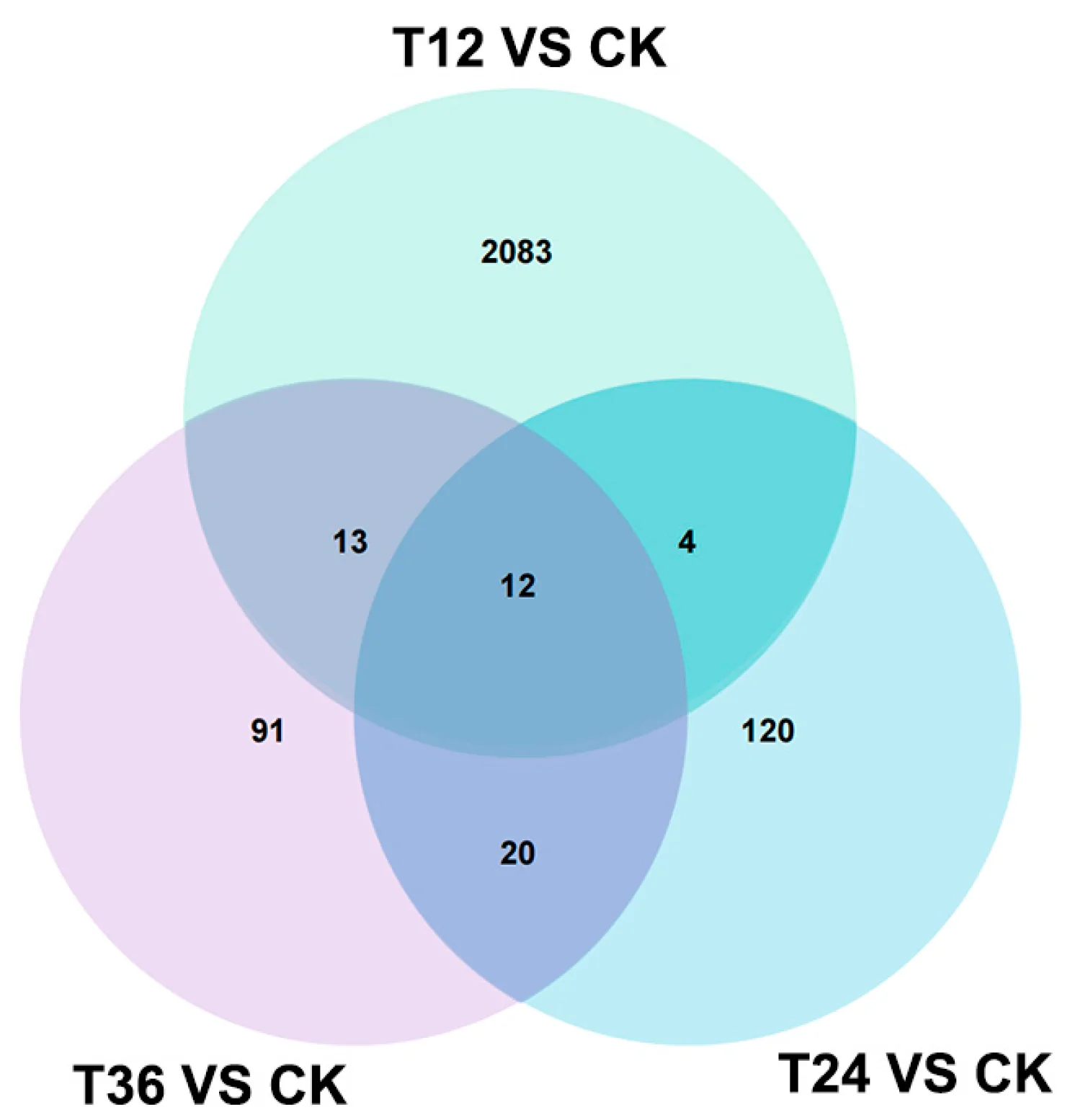
Venn diagram of differentially expressed genes (DEGs) among the T12 vs. CK, T24 vs. CK, and T36 vs. CK comparisons.

A Venn diagram was used to compare the overlap of DEGs among the CK_vs_T12, CK_vs_T24, and CK_vs_T36 comparisons (Figure A2 in Appendix B). The CK_vs_T12 comparison contained the largest number of unique DEGs, with 2083 genes specifically identified at this time point. In contrast, 120 and 91 DEGs were uniquely detected in the CK_vs_T24 and CK_vs_T36 comparisons, respectively. A total of 12 DEGs were shared among all three comparisons, suggesting that these genes may represent core transcriptional responses to treatment (Figure A2 in Appendix B). Analysis of the 12 common differentially expressed genes revealed that nine were significantly upregulated and three were significantly downregulated following imidacloprid treatment. Among the nine upregulated genes, two were annotated as detoxification enzyme genes (c27289.graph_c0 and c58674.graph_c0), two as heat shock protein genes, one as a facilitated trehalose transporter, one as a CAP-Gly domain-containing linker protein, and three as uncharacterized proteins. We prioritized candidate genes for functional validation based on multiple criteria, including their annotation as known detoxification genes, the magnitude of their transcriptional responses, and their potential biological relevance to insecticide metabolism. Representative detoxification genes were then selected for further functional analysis. Overall, these results indicate that the transcriptional response was most pronounced at T12, whereas substantially fewer DEGs were detected at later time points.

### 3.6. Identification and Expression of P450, GST and Esterase Genes

**Figure 4:**
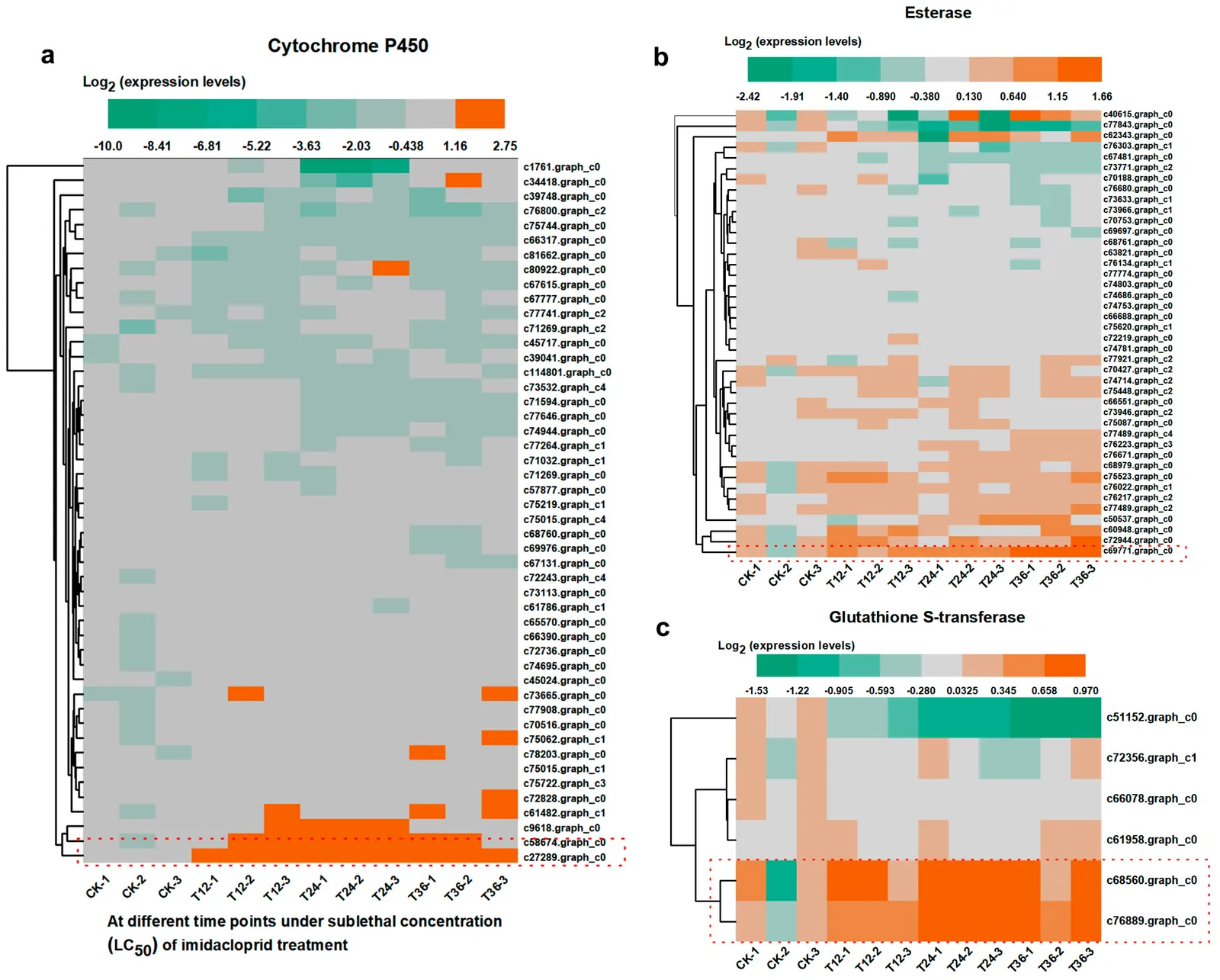
Temporal expression profiles of cytochrome P450 (**a**), carboxylesterase (**b**), and glutathione S-transferase (**c**) genes in response to median lethal (LC_50_) imidacloprid exposure in *Aphis spiraecola* based on transcriptome data.

Transcriptome analysis identified a total of 46 P450 genes, 42 esterase genes, and six GST genes in *A. spiraecola* (Figure 4). Heatmap analysis was conducted to characterize the expression patterns of three detoxification enzyme genes in *A. spiraecola* at different time points under median lethal imidacloprid exposure. Cytochrome P450 genes exhibited distinct expression patterns across the four different time points, with several genes showing increased transcript levels following imidacloprid exposure. A subset of P450 genes, including c27289.graph_c0 and c58674.graph_c0, exhibited higher expression levels in the treated groups than in the CK group, suggesting their potential involvement in the response to imidacloprid stress (Figure 4a).

For esterase genes, most transcripts showed moderate or limited changes across treatments, and the expression of c69717.graph_c0 were induced following imidacloprid exposure, indicating that esterase-mediated detoxification may contribute to the response of *A. spiraecola* to imidacloprid (Figure 4b). Glutathione S-transferase genes also exhibited treatment-responsive expression patterns. Among them, c68560.graph_c0 and c76889.graph_c0 were significantly induced after imidacloprid exposure, especially at T24, suggesting that the two GST genes may be involved in imidacloprid-induced detoxification or oxidative stress responses (Figure 4c).

### 3.7. QPCR Validation of Five Differentially Expressed Genes

**Figure 5:**
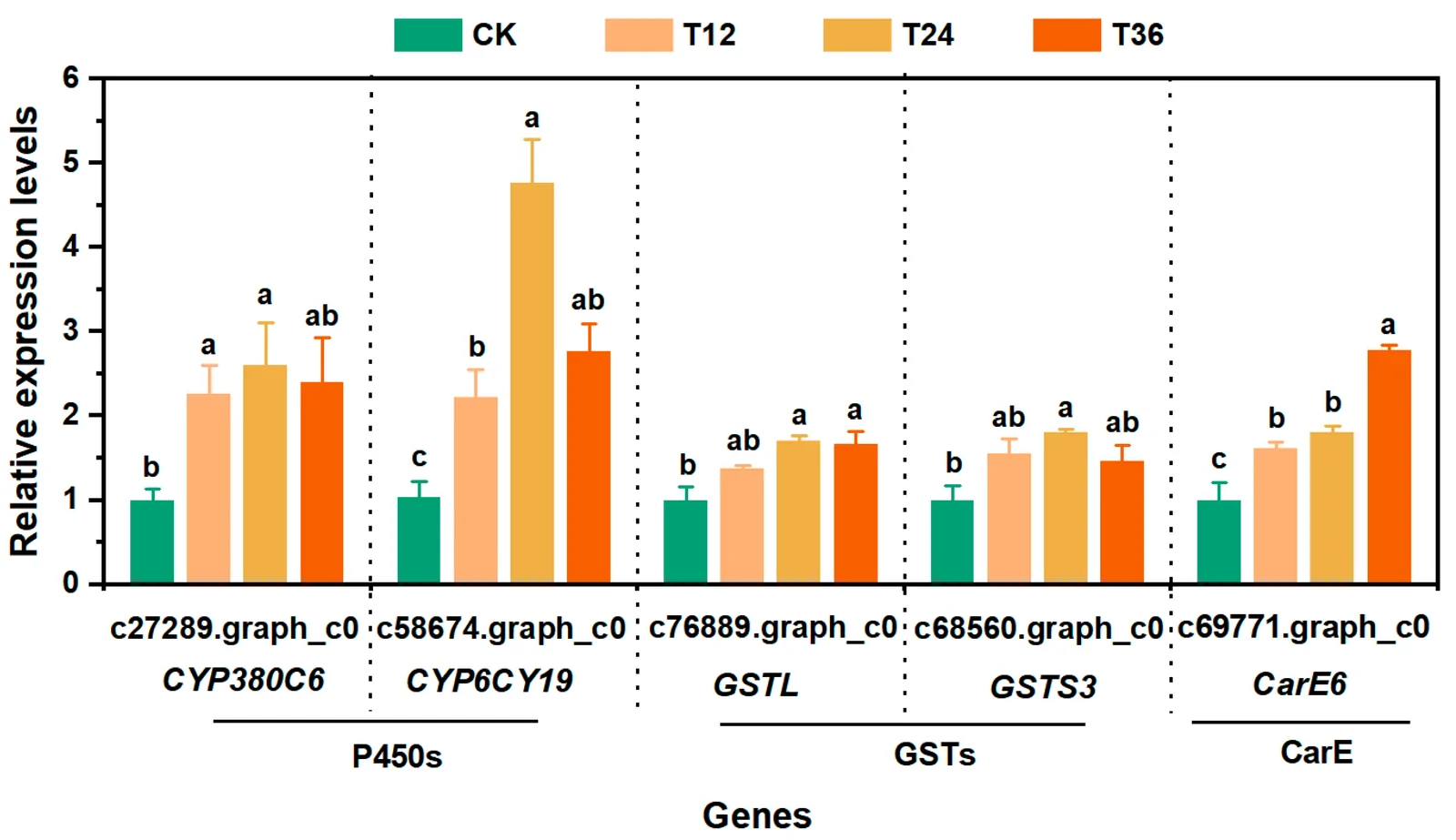
Expression levels of two P450 genes, two GST genes, and one carboxylesterase gene at different time points under median lethal concentration (LC_50_) of imidacloprid treatment, as determined by qPCR. Data are presented as the mean ± SEM. Bars labeled with different lowercase letters indicate significant differences among treatments, as determined by one-way ANOVA followed by Tukey’s multiple comparison test (*p* < 0.05).

Based on the transcriptome data, five differentially expressed genes were selected for further validation by qPCR. These genes included two P450 genes, c27289.graph_c0 and c58674.graph_c0 (hereafter referred to as *CYP380C6* and *CYP6CY19*); two GST genes, c68560.graph_c0 and c76889.graph_c0 (hereafter referred to as *GSTS3* and *GSTL*); and one carboxylesterase gene, c69717.graph_c0 (hereafter referred to as *CarE6*). The qPCR results showed that all five genes were significantly induced following imidacloprid exposure (Figure 5). The *CYP380C6* gene showed significantly higher expression levels at 12 and 24 h after imidacloprid treatment than in the control group (F = 9.01; *df* = 3, 8; *p* = 0.006). The expression of *CYP6CY19* was significantly upregulated at 12, 24, and 36 h after imidacloprid exposure compared with the control, with the highest expression level observed at 24 h (F = 4.43; *df* = 3, 8; *p* = 0.041). *GSTS3* showed significantly increased expression at 24 and 36 h after imidacloprid exposure compared with the control (F = 4.00; *df* = 3, 8; *p* = 0.045). The expression of *GSTL* was significantly increased at 24 h after imidacloprid treatment relative to the control (F = 10.24; *df* = 3, 8; *p* = 0.004). No significant differences were observed among the three post-treatment time points for *GSTL* and *GSTS3*. The expression of *CarE6* was significantly upregulated at 12, 24, and 36 h after imidacloprid exposure compared with the control, with the highest expression level detected at 36 h (F = 37.49; *df* = 3, 8; *p* < 0.001).

### 3.8. Determination of RNA Interference Efficiency

**Figure A3:**
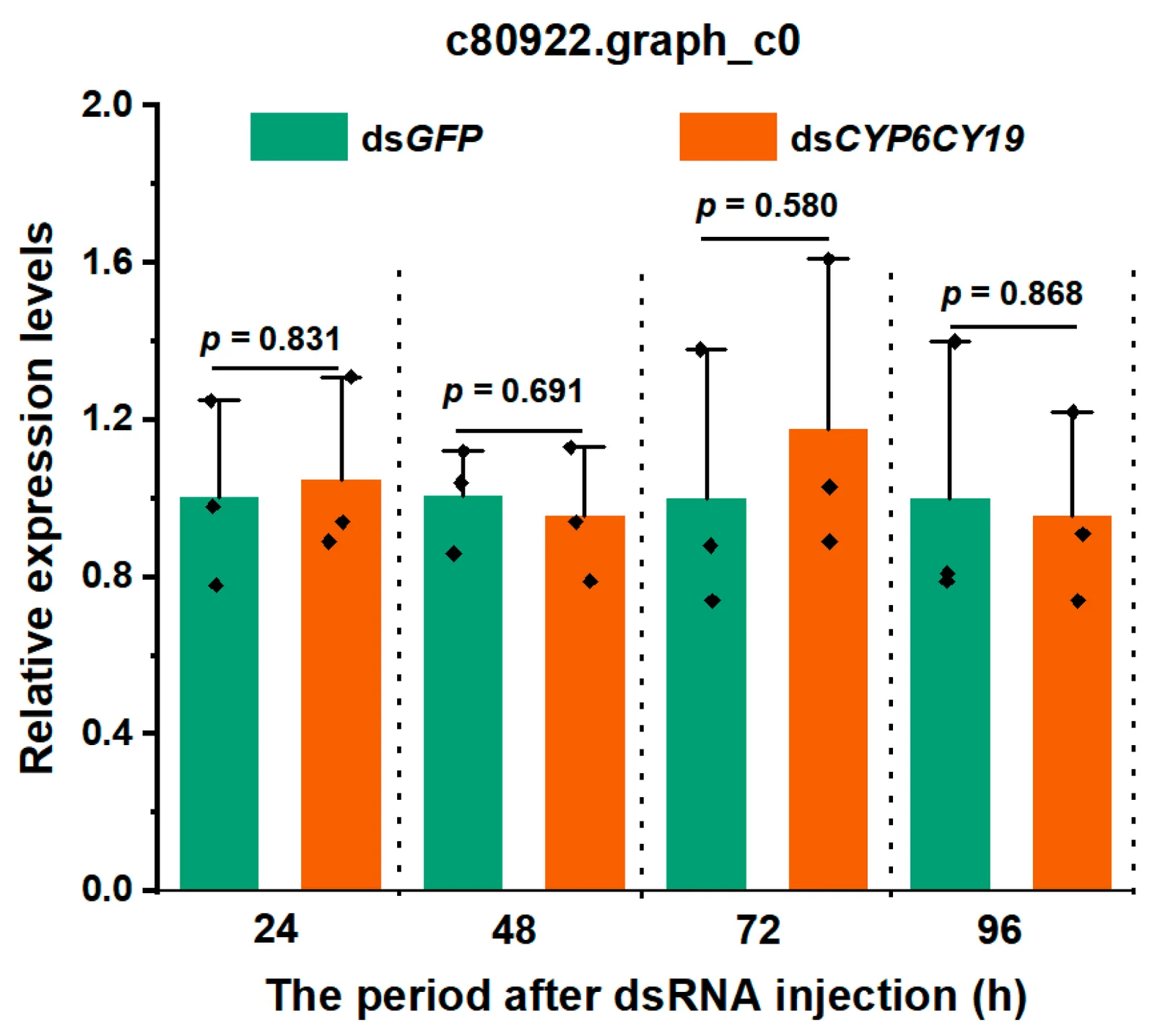
Effect of ds*CYP6CY19* injection on the expression level of c80922.graph_c0.

Sequence homology analysis identified only one P450 target gene (*CYP6CY19*) with a highly similar homolog gene, c80922.graph_c0. To assess whether RNAi targeting *CYP6CY19* affected the expression of c80922.graph_c0, its transcript level was examined following dsRNA treatment. Although expression of *CYP6CY19* was significantly reduced at 48 h and 72 h after dsRNA injection, no significant change in c80922.graph_c0 expression was detected compared with the ds*GFP* control (*t*-test, *p* > 0.05, Figure A3 in Appendix B). These results suggest that the dsRNA treatment specifically silenced the intended target gene without detectable off-target effects on the closely related homolog examined.

**Figure 6:**
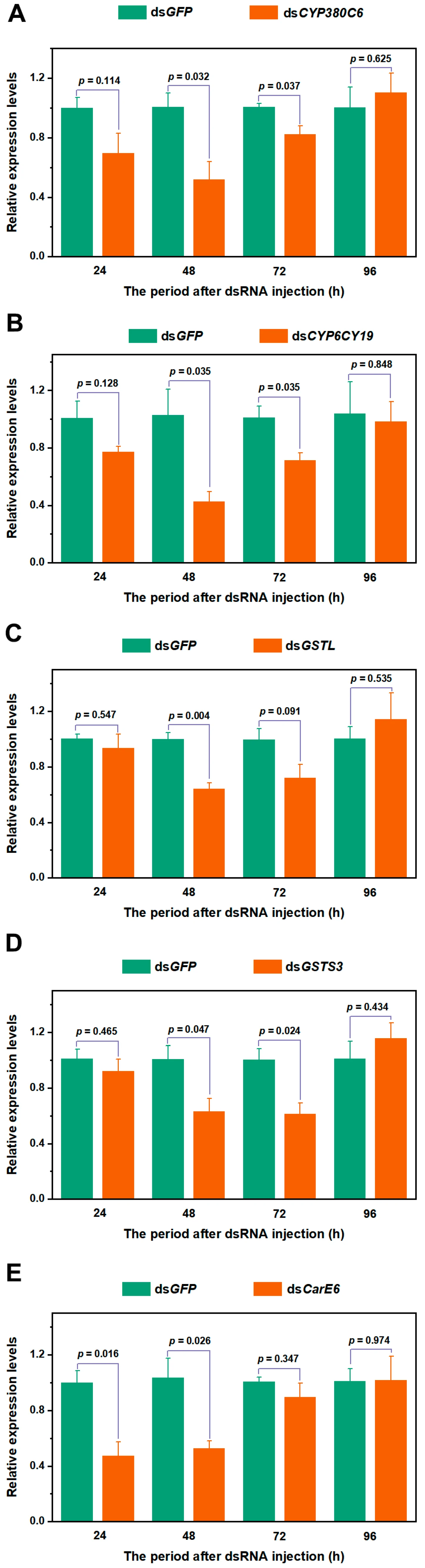
Effects of injections with ds*CYP380C6* (**A**), ds*CYP6CY19* (**B**), ds*GSTL* (**C**), ds*GSTS3* (**D**), and ds*CarE6* (**E**) on the expression levels of their corresponding target genes at different time points. Data are presented as the mean ± SEM from three independent replicates. *p* values were determined using Student’s *t*-test.

*A. spiraecola* were collected at 24, 48, 72, and 96 h after dsRNA injection to evaluate the gene-silencing efficiency at different time points. For *CYP6CY19*, the dsRNA fragment was 369 bp in length and corresponded to nucleotides of the coding sequence (462–830 bp). The dsRNA fragments of *CYP380C6*, *GSTL*, *GSTS3* and *CarE6* were 278, 201, 313 and 414 bp, respectively (Table A2 in Appendix A). The RNA interference efficiency of *CYP380C6*, *CYP6CY19*, *GSTS3*, *GSTL*, and *CarE6* in *A. spiraecola* was then evaluated at different time points after dsRNA injection (Figure 6). Compared with the ds*GFP* control, the transcript level of *CYP380C6* was significantly reduced at 48 h and 72 h after ds*CYP380C6* injection, with interference efficiencies of 48.67% (*t*-test, *p* = 0.032) and 18.33% (*t*-test, *p* = 0.037), respectively (Figure 6A). However, no significant difference in *CYP380C6* expression was observed at 24 h (*t*-test, *p* = 0.114) or 96 h (*t*-test, *p* = 0.625) post-injection. The expression of *CYP6CY19* was significantly decreased at 48 h and 72 h after ds*CYP6CY19* injection, with reductions of 60.12% and 29.79%, respectively (Figure 6B). The silencing efficiency of *GSTL* peaked at 48 h after dsRNA injection, reaching 35.67% (*t*-test, *p* = 0.004), and the expression of *GSTL* did not differ significantly from that in the ds*GFP* control at 24 h (*t*-test, *p* = 0.547), 72 h (*t*-test, *p* = 0.091), or 96 h (*t*-test, *p* = 0.535) after ds*GSTL* injection (Figure 6C). For *GSTS3*, transcript levels were also significantly reduced at 48 h (*t*-test, *p* = 0.047) and 72 h (*t*-test, *p* = 0.024) after ds*GSTS3* injection, with silencing efficiencies of 37.67% and 39.00%, respectively (Figure 6D). Injection of ds*CarE6* significantly suppressed *CarE6* expression at 12 h (*t*-test, *p* = 0.016) and 24 h (*t*-test, *p* = 0.026) post-injection compared with the ds*GFP* control, with reductions of 52.67% and 51.00%, respectively.

### 3.9. CYP6CY19 and CarE6 Affect the Susceptibility of A. spiraecola to Imidacloprid

**Figure 7:**
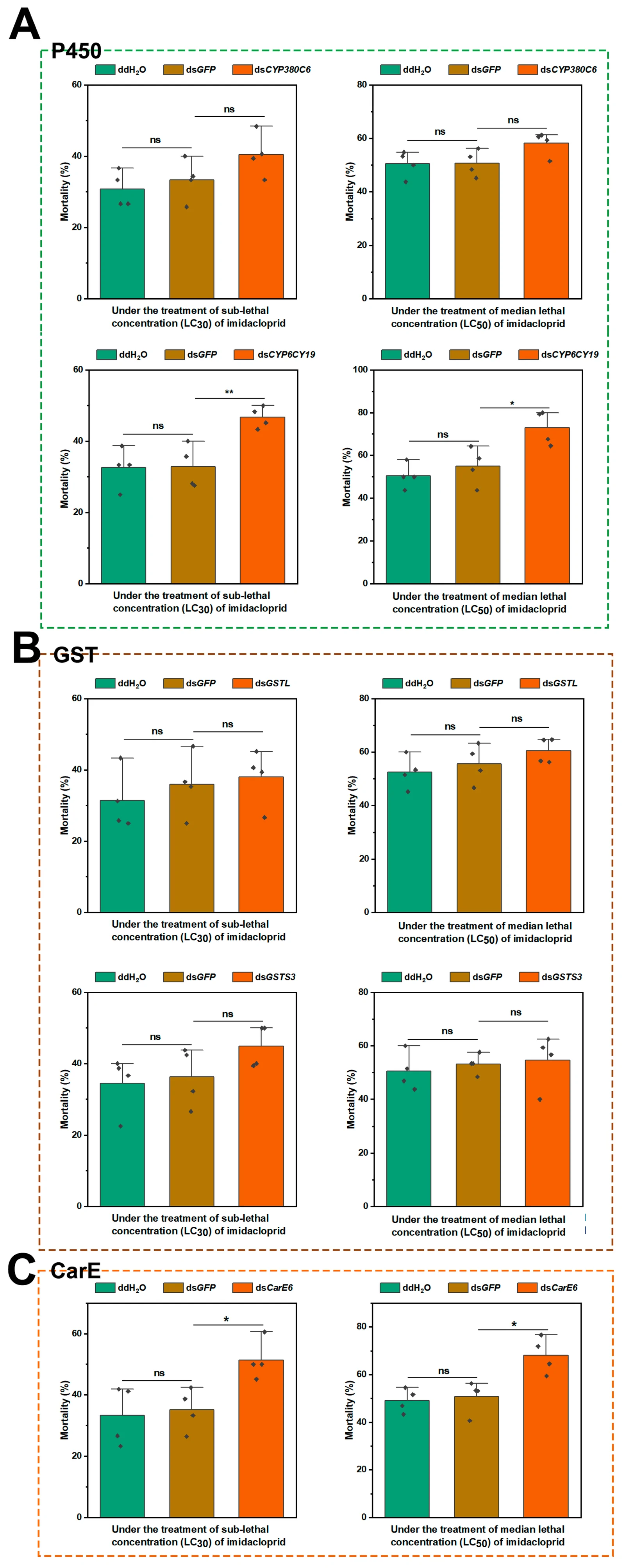
Mortality (%) of *Aphis spiraecola* injected with ds*CYP380C6* (**A**), ds*CYP6CY19* (**A**), ds*GSTL* (**B**), ds*GSTS3* (**B**), or ds*CarE6* (**C**) following treatment with sublethal concentrations of imidacloprid (LC_30_ or LC_50_). A single asterisk (*) and double asterisks (**) denote significant differences compared with the control group at *p* < 0.05 and *p* < 0.01, respectively. “ns” indicates no significant difference.

To further investigate the expression of these five upregulated genes, we performed RNAi-mediated functional validation. Under sublethal imidacloprid exposure, the mortality of *A. spiraecola* injected with ds*GFP* was not significantly different from that of aphids injected with ddH_2_O, indicating that ds*GFP* injection did not affect aphid susceptibility to imidacloprid (Figure 7). For the P450 genes, the knockdown of *CYP380C6* did not significantly alter the mortality of *A. spiraecola* under either LC_30_ or LC_50_ imidacloprid treatment compared with the ds*GFP*-injected control (Figure 7A). In contrast, the knockdown of *CYP6CY19* significantly increased aphid mortality following exposure to sublethal concentrations of imidacloprid (*t*-test, LC_30_: *p* = 0.006; LC_50_: *p* = 0.023), suggesting that *CYP6CY19* contributes to the tolerance of *A. spiraecola* to imidacloprid (Figure 7A).

For GST genes, the knockdown of *GSTL* (*t*-test, LC_30_: *p* = 0.741; LC_50_: *p* = 0.302) or *GSTS3* (*t*-test, LC_30_: *p* = 0.142; LC_50_: *p* = 0.792) did not significantly affect mortality of *A. spiraecola* under LC_30_ or LC_50_ imidacloprid exposure compared with the ds*GFP* control (Figure 7B). When exposed to sublethal concentrations of LC_30_ (*t*-test, *p* = 0.014) or LC_50_ (*t*-test, *p* = 0.016) in imidacloprid, the mortality of *A. spiraecola* injected with ds*CarE6* was significantly higher than that of the ds*GFP*-injected control group (Figure 7C).

## 4. Discussion

Chemical insecticides remain the primary strategy for controlling *A. spiraecola* because of their rapid lethal effects and high efficacy in suppressing population growth. Interestingly, insecticide exposure not only causes direct mortality in *A. spiraecola* but may also substantially affect aphid metabolic response, reproductive performance, and the development of insecticide resistance [11,30]. Imidacloprid, a representative neonicotinoid insecticide that targets insect nicotinic acetylcholine receptors, has been widely used against aphids and other piercing–sucking pests. Elucidating how aphids respond to imidacloprid at the enzymatic, transcriptional, and functional levels is essential for optimizing field management strategies. In the present study, we found that the enzyme activities of P450, GST and CarE were significantly increased after exposure to median lethal concentration of imidacloprid, and the toxicity of imidacloprid against *A. spiraecola* was significantly increased after application of the inhibitors of PBO and TPP. Transcriptome analysis revealed that LC_50_ concentrations of imidacloprid treatment induced time-dependent changes in gene expression at 12, 24, and 36 h. Among the imidacloprid-responsive genes, *CYP380C6*, *CYP6CY19*, *GSTS3*, *GSTL* and *CarE6* were significantly upregulated, as confirmed by transcriptome and qPCR analysis. RNAi-based functional assays demonstrated that the silencing of *CYP6CY19* and *CarE6* genes significantly increased the susceptibility of *A. spiraecola* to imidacloprid, whereas the silencing of *CYP380C6* and the two GST genes did not significantly affect susceptibility, suggesting that imidacloprid exposure activates a broad detoxification response in *A. spiraecola*, but only a subset of inducible detoxification genes functions acts as major determinants of imidacloprid susceptibility.

Transcriptome analysis is a powerful approach for characterizing transcript profiles, elucidating gene expression regulation, and annotating potential biological functions [31]. In this study, we found that imidacloprid exposure induced a dynamic transcriptional response in *A. spiraecola* at 12, 24, and 36 h. The number of differentially expressed genes was highest at the early stage of treatment, indicating that *A. spiraecola* rapidly activates broad transcriptional reprogramming after insecticide challenge. Functional enrichment analyses further suggested that imidacloprid exposure affected multiple biological processes, including protein processing in the endoplasmic reticulum, oxidative phosphorylation, carbon metabolism, fatty acid metabolism, ribosome-related processes, and xenobiotic detoxification pathways. Moreover, we identified 46 P450 genes, 42 esterase genes, and six GST genes in *A. spiraecola* based on the transcriptome dataset. Temporal expression of these genes at different time points suggests that imidacloprid exposure does not activate a single detoxification route, but instead induces a coordinated response involving phase I functionalization and phase II conjugation. Comparable transcriptional responses that vary according to exposure time, insecticide dose, and species have been reported in other insect species. In *Aphis craccivora*, 38 P450 genes and 10 GST genes were identified through transcriptomic analysis, and *CYP6DA2*, *CYP4CJ1*, *CYP380C6* and several GST genes were responsive to imidacloprid exposure, although their expression patterns and functional contributions differed [20]. Amplification and overexpression of *CYP6CY3* of *Myzus persicae* were strongly associated with neonicotinoid resistance, demonstrating that transcriptional activation of a specific P450 gene can represent a central mechanism of aphid adaptation to neonicotinoid selection pressure [32]. In *Aphis glycines*, *CYP6CY7* was identified from transcriptomic resources and functionally linked to imidacloprid detoxification [23]. *CYP6A14-1*, *CYP307A1*, *GST1-1-1*, and *COE2* from *Sitobion avenae* were overexpressed in an imidacloprid-resistant strain and were implicated in imidacloprid resistance [19]. *Leptinotarsa decemlineata*, an imidacloprid-resistant beetle, over-expresses multiple detoxification-related genes, including P450s, esterases, GSTs, UGTs, and ABC transporters, suggesting that complex metabolic networks, rather than a single enzyme family, can underlie imidacloprid resistance [33]. Through transcriptome analysis and qPCR validation, we found that the *CYP380C6*, *CYP6CY19*, *GSTS3*, *GSTL*, and *CarE6* genes were induced expression after exposure to imidacloprid in *A. spiraecola*. The significant increases in P450, GST, and CarE activities after treatment with different concentrations of imidacloprid further confirmed that imidacloprid activates multiple detoxification systems in *A. spiraecola*. Moreover, the application of the synergists PBO and TPP significantly enhanced the toxicity of imidacloprid against *A. spiraecola*, whereas DEM had no significant effect. These results suggest that P450- and carboxylesterase-mediated detoxification may contribute to the susceptibility of *A. spiraecola* to imidacloprid, whereas GST-mediated detoxification may play a limited role under the tested conditions.

To further clarify the roles of these five genes in imidacloprid susceptibility, RNAi was employed to functionally validate their contributions to insecticide tolerance in *A. spiraecola*. Cytochrome P450s are among the most important metabolic enzymes involved in insecticide detoxification, and many cases of neonicotinoid resistance in hemipteran insects have been associated with P450 overexpression. In this study, both *CYP380C6* and *CYP6CY19* were significantly upregulated after imidacloprid exposure; however, RNAi produced contrasting phenotypes. The knockdown of *CYP6CY19* significantly increased the susceptibility of *A. spiraecola* to imidacloprid, whereas interference of *CYP380C6* did not significantly affect the susceptibility. These results indicated that *CYP6CY19* is more likely to function as a key gene for imidacloprid susceptibility, and functional redundancy among P450 paralogs or compensatory induction of other detoxification genes may also mask the effect of *CYP380C6* silencing. Such a difference is biologically plausible because P450 genes often display strong substrate specificity, tissue-specific expression, and functional divergence, even within the same CYP family. Similar phenomena have been reported in other insects. In *A. craccivora*, *CYP6DA2*, *CYP4CJ1* and *CYP380C6* were induced by imidacloprid, but RNAi significantly increased susceptibility mainly after silencing *CYP6DA2* and *CYP380C6*, indicating that not all induced P450 genes contribute equally to imidacloprid tolerance [20]. *CYP9Z82*, *CYP4BD4*, *CYP6NR1*, and *CYP345J1* were among the most highly upregulated P450 genes in *Rhynchophorus ferrugineus*. Oral delivery of dsRNA effectively knocked down the expression of *CYP9Z82*, *CYP6NR1*, and *CYP345J1*. Functional assays further showed that silencing *CYP345J1* and *CYP6NR1* significantly reduced the survival of *R. ferrugineus* exposed to imidacloprid, indicating that these two P450 genes may contribute importantly to imidacloprid tolerance [34]. RNAi-mediated knockdown of three P450 genes (*CYP6BQ15*, *CYP4Q3* and CYP4Q7) that were significantly upregulated in imidacloprid-resistant beetle strains showed that silencing *CYP4Q3* significantly increased the susceptibility of resistant beetles to imidacloprid [33]. The knockdown of *CYP6AY1*, *CYP6ER1*, *CYP4CE1* and *CYP6CW1* in *N. lugens* [35,36,37], *CYP6DP1* and *CYP6DP2* of *T. vaporariorum* [38], *CYP6DP3* and *CYP6CM1* in *B. tabaci* [39,40], *CYP6CY7* in *A. glycines* [23], and *CYP6CY3* in *M. persicae* [41], significantly increased imidacloprid susceptibility, further supporting gene-specific P450 involvement in insects.

Carboxylesterases are a large group of hydrolytic enzymes involved in endogenous ester metabolism and xenobiotic detoxification. In insects, they are known for their roles in resistance to organophosphates, carbamates, and pyrethroids. In *M. persicae*, the recombinant E4 carboxylesterase was shown to degrade malathion, an organophosphate insecticide, and carbaryl, a carbamate insecticide [42]. Carboxylesterase-mediated pyrethroid detoxification has also been functionally demonstrated in *Helicoverpa armigera*, in which *CarE001A* and *CarE001H* contributed to the detoxification of pyrethroids [43]. In addition to these ester-containing insecticides, carboxylesterases may also contribute to tolerance or resistance to other insecticide classes through hydrolysis, sequestration, or indirect metabolic regulation. For instance, *COE2* in *S. avenae* was overexpressed in an imidacloprid-resistant strain, and RNAi feeding targeting *COE2* increased imidacloprid susceptibility [19], suggesting that carboxylesterases may participate in neonicotinoid tolerance in some insects. In *Bactrocera dorsalis*, *BdCarE20* silencing could significantly increase the toxicity of spinosad, abamectin, cyantraniliprole, imidacloprid, and λ-cyhalothrin in the SS strain, and in the MR strain, *BdCarE33* had an important role in malathion toxicity [44]. In the present study, *CarE6* was significantly up-regulated after imidacloprid exposure, and the application of TPP, a carboxylesterase inhibitor, significantly enhanced the toxicity of imidacloprid against *A. spiraecola*, suggesting that *CarE6* may contribute to imidacloprid tolerance in this aphid. RNAi-mediated silencing of *CarE6* significantly increased the susceptibility of *A. spiraecola* to imidacloprid. These results suggests that *CarE6* is not only transcriptionally responsive to imidacloprid but also functionally involved in imidacloprid tolerance.

Glutathione S-transferases are multifunctional phase II detoxification enzymes that catalyze the conjugation of reduced glutathione to a wide range of electrophilic substrates, thereby increasing their solubility and facilitating detoxification. Numerous studies have shown that insect GSTs facilitate adaptation to chemical stress through direct metabolism, sequestration of xenobiotics, and mitigation of xenobiotic-induced oxidative stress [45]. In *Plutella xylostella*, *GSTu1* was confirmed to contribute to chlorantraniliprole resistance by directly degrading this insecticide [46]. A GST gene cluster was strongly associated with lambda-cyhalothrin resistance, and RNAi-mediated silencing of *GST_119*, or co-silencing of *GST_119* and *GST_121*, significantly increased larval susceptibility to lambda-cyhalothrin in *Helicoverpa armigera* [47]. Several GST genes from *N. lugens*, including *NlGSTs1, NlGSTs2*, *NlGSTe1*, and *NlGSTm1*, were shown to contribute to imidacloprid resistance, potentially by metabolizing secondary products generated during phase I detoxification [22]. Silencing of *GST1-1-1* by dsRNA feeding resulted in increased susceptibility of *S. avenae* to imidacloprid [19]. Interestingly, *GSTS3* and *GSTL* were significantly up-regulated after imidacloprid exposure, and total GST activity increased after imidacloprid treatment in this study. However, the application of DEM, a glutathione S-transferase inhibitor, could not significantly affect the toxicity of imidacloprid against *A. spiraecola*, and the knockdown of *GSTS3* or *GSTL* did not significantly alter the susceptibility of *A. spiraecola* to imidacloprid. These results suggest that *GSTS3* and *GSTL* may participate in the general stress response induced by imidacloprid exposure, but they are unlikely to be the primary determinants of imidacloprid susceptibility in *A. spiraecola*. Among the GST genes in *A. craccivora*, only *sigma1* was up-regulated in the JY field population were induced by imidacloprid, but functional evidence highlighted P450 genes as more important determinants of imidacloprid susceptibility [20]. Interestingly, we identified one GST gene (c51152.graph_c0) that was consistently downregulated relative to the controls. Although our functional analyses focused primarily on upregulated detoxification genes, the downregulation of this GST may also have biological significance, potentially reflecting metabolic reallocation, negative feedback regulation, pathway-specific suppression, or an indirect response to imidacloprid exposure. Its specific role in the response to imidacloprid remains unclear and will be investigated in future studies.

Overall, our results indicate that P450 and *CarE* play important roles in the response of *A. spiraecola* to imidacloprid exposure. P450s and carboxylesterases may act jointly rather than independently in insecticide detoxification. In imidacloprid-resistant *S. avenae*, both enzyme systems were elevated, PBO and TPP each enhanced imidacloprid toxicity, and silencing of P450- and carboxylesterase-related genes increased insecticide susceptibility, supporting concurrent contributions of multiple metabolic pathways to resistance [19]. Similar patterns have been reported in *Aphis gossypii* and *A. glycines* [48,49]. These findings suggest that simultaneous inhibition of P450- and carboxylesterase-mediated detoxification may produce additive or synergistic effects. Future studies should evaluate combined PBO and TPP treatments to determine whether these pathways cooperatively contribute to imidacloprid tolerance in the apple aphid. Moreover, our findings identify *CYP6CY19* and *CarE6* as key contributors to imidacloprid tolerance in *A. spiraecola* and as potential molecular targets for resistance management. Selective inhibition of either enzyme may therefore provide a strategy for developing resistance-breaking synergists to enhance insecticide efficacy. However, current synergists such as PBO and TPP broadly inhibit detoxification enzyme classes rather than specific isoforms. Future studies should therefore characterize *CYP6CY19* and *CarE6* biochemically, identify isoform-selective inhibitors, and evaluate their efficacy and non-target safety under field-relevant conditions.

## 5. Conclusions

In conclusion, this study demonstrates that LC_50_ imidacloprid exposure elicits a complex detoxification response in *A. spiraecola*, including the increased enzymatic activities of P450s, GSTs, and CarEs, accompanied by transcriptional induction of *CYP380C6*, *CYP6CY19*, *GSTS3*, *GSTL*, and *CarE6*. Functional validation using RNAi revealed that only *CYP6CY19* and *CarE6* significantly influenced imidacloprid susceptibility. The current findings enhance our understanding of the mechanisms underlying insect responses to imidacloprid and provide valuable insights for the rational use of this insecticide in aphid management. Moreover, integrating RNAi-based strategies targeting key detoxification genes could further enhance imidacloprid efficacy and contribute to the development of synergist-assisted, precision-targeted pest control approaches. This study specifically investigated the effects of silencing two detoxification genes on aphid susceptibility to imidacloprid. Future research should aim to heterologously express and purify *CYP6CY19* and *CarE6*, followed by in vitro metabolism assays and LC-MS/MS-based detection of imidacloprid metabolites, in order to directly elucidate the catalytic roles of these enzymes in insecticide detoxification.

## Figures and Tables

**Table 1:** Toxicity of imidacloprid against *Aphis spiraecola.*

Slope ± SE	LC_20_ (95% Confidence Limit) (mg·L^−1^)	LC_30_ (95% Confidence Limit) (mg·L^−1^)	LC_50_ (95% Confidence Limit) (mg·L^−1^)	Correlation Coefficient
3.21 ± 0.42	7.45 (6.37–8.70)	9.35 (8.03–12.21)	13.61 (11.33–17.74)	0.95

**Table 2:** Summary of transcriptome analysis in *Aphis spiraecola*.

Samples	Read Number	Base Number	GC Content	Q_30_
CK_1	25,247,054	7,555,134,642	38.38%	90.54%
CK_2	23,222,672	6,947,707,940	37.46%	90.42%
CK_3	23,972,351	7,174,891,268	38.55%	90.63%
T12_1	25,274,858	7,567,395,378	39.66%	90.23%
T12_2	21,702,355	6,490,721,134	39.21%	90.07%
T12_3	30,335,523	9,079,920,522	39.56%	90.41%
T24_1	24,238,515	7,252,580,824	38.79%	90.70%
T24_2	27,308,073	8,171,899,914	38.82%	90.61%
T24_3	22,761,635	6,809,401,690	38.71%	90.59%
T36_1	23,235,126	6,948,500,898	38.63%	90.36%
T36_2	22,038,245	6,591,300,180	38.81%	90.65%
T36_3	28,177,216	8,433,781,110	39.10%	90.40%

## Data Availability

Data are contained within the article.

## Appendix A

**Table A1:** Length distribution of transcript and unigene in *Aphis spiraecola.*

Length Range (bp)	Transcript	Unigene
200–300	12,150 (10.82%)	11,347 (19.09%)
300–500	11,648 (10.38%)	9287 (15.62%)
500–1000	22,216 (19.79%)	14,694 (24.72%)
1000–2000	26,062 (23.22%)	12,236 (20.59%)
>2000	40,168 (35.78%)	11,874 (19.98%)
Total Number	112,250	59,440
Total Length	226,795,604	79,947,644
N50 Length	3408	2412
Mean Length	2020.45	1345.01

**Table A2:** The primers for quantitative PCR and RNA interference.

Name	Forward Primer	Reverse Primer	Product Size (bp)
Q_CYP380C6	GGACCGCCGTCATACCCAAT	TCACCACCATAGCTGCTGAACA	104
Q_CYP6CY19	CGCCGGTGCTGAACCTGTAT	GCTGACGCAGTTTGTCTTGGAT	89
Q_GSTL	GCGAACAATGGCCTACACTCA	TTCCCAATCATCACTACCAGCT	146
Q_GSTS3	ATGGAAAGTTGTCCTGGGCTGA	TGCAGGTCTCTTGGCGATCC	164
Q_CarE6	CGGATCTGTGTACCAGCCTGAT	CCTCCAGCCGACATTCCACTAA	225
RNAi_CYP380C6	TTAAATATTCTGCGGGCTGG	CACTCTAGCCCTGTGCCAAT	278
RNAi_CYP6CY19	GTATGGCCAGGTCAACGACT	ACATCGTTTCTGGTTACGCC	369
RNAi_GSTL	TTACTGCTTTGGCTGAACCC	CGGTTTGAGTGTAGGCCATT	201
RNAi_GSTS3	ATGGCCATCAATTAAACCCA	TCCGTCATTTTCATCCACAA	313
RNAi_CarE6	TAGCAGCAGATGTACCGTGG	CTGCATGACTTGCTCCGTAA	414
RNAi_GFP	GTGTTCAATGCTTTTCCCGT	CAATGTTGTGGCGAATTTTG	356

The T7 adapter sequence (GGATCCTAATACGACTCACTATAGG) was added onto the 5′ end of the RNA interference primer.
